# The impact of COVID-19 pandemic in urology practice, assistance and residency training in a tertiary referral center in Brazil

**DOI:** 10.1590/S1677-5538.IBJU.2020.0761

**Published:** 2021-01-10

**Authors:** Antonio Rebello Horta Gorgen, Johanna Ovalle Diaz, Aline Gularte Teixeira da Silva, Artur Paludo, Renan Timoteo de Oliveira, Patric Machado Tavares, Tiago Elias Rosito

**Affiliations:** 1 Hospital de Clínicas de Porto Alegre Serviço de Urologia Porto AlegreRS Brasil Serviço de Urologia, Hospital de Clínicas de Porto Alegre, Porto Alegre, RS, Brasil; 2 Hospital de Clínicas de Porto Alegre Grupo de Urologia Reconstrutiva e Infantil Porto AlegreRS Brasil Grupo de Urologia Reconstrutiva e Infantil, Hospital de Clínicas de Porto Alegre, Porto Alegre, RS, Brasil; 3 Universidade Federal do Rio Grande do Sul Departamento de Ginecologia e Obstetrícia Porto AlegreRS Brasil Departamento de Ginecologia e Obstetrícia, Universidade Federal do Rio Grande do Sul, Porto Alegre, RS, Brasil; 4 Universidade Federal do Rio Grande do Sul Departamento de Cirurgia Porto AlegreRS Brasil Departamento de Cirurgia, Universidade Federal do Rio Grande do Sul, Porto Alegre, RS, Brasil

## INTRODUCTION

The COVID-19 global pandemic has significantly impacted healthcare systems throughout the World and resources were reallocated to combat the pandemic. Urology practice was deeply affected, with most guidelines publishing recommendations to postpone most of elective surgeries (1).

To this day, Brazil is the second most affected country with over 3.3 million people COVID-19 cases and over 107.000 deaths. Since the end of March, a partial lockdown has been conducted in most cities even in those without any cases or deaths yet, and, four months later, there is still a high number of daily new cases.

Hospital de Clínicas de Porto Alegre is one of the largest in Brazil, doing over 49.000 surgeries in the last year and over 567.000 outpatient consultations. Our patients are 95% from the public healthcare system, receiving patients from all cities in our state, and 5% from private care, so we can consider our hospital a representative part of the public system. Our Urology department is one of the largest surgical departments at our hospital, performing over 5.000 surgeries every year and is responsible for over 30% of the urological high complexity surgeries in our region.

This year, however, the state secretary of health designed our hospital as the main referral center for COVID-19 patients in our state. Therefore, all departments, including Urology, made a contingency plan to reallocate resources and postpone elective surgeries.

This study aims to analyze the impact in urology practice through the variation in outpatient clinics, urodynamics exams, and surgeries during the pandemic months compared to the same period of previous years and its effect on residency training compared to regular years.

## MATERIAL AND METHODS

This study was approved by our Ethics Committee with the IRB number 31645020.5.0000.5327.

We performed a prospective analysis of the total volume of urological outpatient and inpatient consultations, urodynamics exams, hospitalizations and surgeries from April to July, 2020 and compared to the average number of cases in the same months of previous years (2019 and 2018) in a university hospital in southern Brazil.

Our hospital developed a contingency plan with four groups depending on the intensive care unit (ICU) occupation and COVID-19 cases. Each department then developed a specific contingency plan regarding the outpatient clinic, elective surgeries, and urgent surgeries. Since the end of March, the Urology team has evaluated all scheduled ambulatory and elective surgery and postponed them whenever possible. Also, urology residents were reallocated to backup for non-urological activities.

Our contingency plan during the COVID-19 pandemic divided the outpatient clinic, exams, and surgeries into levels of urgency and/or gravity (Table-1). Most new appointments were postponed, except for oncology or urgent appointments (the healthcare is regulated by the city health secretary; urgent consultations are defined by the primary care physician). Routine appointments were all canceled, except for high-risk oncology, postoperative appointments, patients with dressings or catheters, active infections, or risk of death or organ injury. Urodynamics were also canceled except for specific cases defined by the urology team that could affect the patient's health in the short-term. Surgeries were divided into four groups:

**Table 1:** COVID-19 Contingency Plan for the Urology Department of Hospital de Clínicas de Porto Alegre.

Group	Group 1 Surgeries	Group 2 Surgeries	Group 3 Surgeries	Group 4 Surgeries
Definition	Urgent surgeries; surgeries with imminent risk of death or organ injury	High-risk oncology surgeries; others procedures with infections associated	Low-risk oncology surgeries; Stones with catheters	Other procedures without infections associated
Examples	Obstructive pyelonephritis, renal abscess, acute urinary retention, Fournier gangrene, priapism, penile fracture, testicular torsion	Radical cystectomies, high-risk radical nephrectomy (T2 or higher), high-risk radical prostatectomy, orchiectomy, nephroureterectomy, lymph node dissections Pyeloplasty, urethroplasty, ureteral reimplant, and simple prostatectomy, in patients with recurrent urinary tract infection	Low-risk radical prostatectomy, low risk partial or radical nephrectomy (T1), prostate biopsies, transurethral bladder resection; Obstructive stones with double J stent or nephrostomies	Reconstructive, incontinence, pediatric, andrology, benign prostate hyperplasia and other procedures without recurrent urinary infection
Plan	Should be operated	Should be operated as long as hospital beds and/or ICU are available	Should be operated during early phase* hospital contingency plan	Should be postponed

* Early phase defined by the hospital direction considering a combination of factors such as the number of COVID-19 patients and ICU occupation.

Group 1 - Urgent surgeries such as obstructive pyelonephritis, priapism, penis fracture, testicle torsion, Fournier gangrene, or surgeries with imminent risk of death. These surgeries would still be done during all the COVID-19 pandemic.

Group 2 - High-risk oncology surgeries such as radical cystectomies, radical nephrectomy (T2 or higher), high-risk radical prostatectomy, orchiectomies, nephroureterectomies, and lymph node dissections for high-risk cancers. These surgeries would still be done as long as hospital beds and ICU were available.

Group 3 - Low-risk oncology surgeries such as partial nephrectomy, low or intermediate-risk prostate cancer, and prostate biopsies and stones with double J stent or nephrostomy. These surgeries would be done as long as the hospital contingency plan was low.

Group 4 - Reconstructive, incontinence, andrology, endoscopic and pediatric procedures. These surgeries would be postponed unless infection or risk of organ injury were assessed.

The outpatient clinic was divided into new or routine (including postoperative) appointments. New appointments are subdivided into General Urology, Oncology, Transplantations, and Pediatric Urology. In the postoperative and routine appointments, oncology is merged with general urology (general urology, transplantations, and pediatric urology) appointments.

Exams are exclusively urodynamics. Other exams, such as prostate biopsy and retrograde pyelography, were included in the procedure section (because these exams are done at our hospital in the operating room and, as such, are classified as procedures).

Surgeries were divided into elective surgeries, urgent surgeries, kidney transplantations, and procedures under local anesthesia. Elective surgeries were analyzed based on the major's urology subspecialties: Oncology, Endourology, Reconstructive/Pediatrics, Andrology, Female/Incontinence, and General Urology.

Inpatient variables analyzed included monthly hospitalization rates, inpatient consultations, emergency department consultations, average length of stay, and mortality rate.

In our urology residency program, all high complexity surgeries are mainly performed by residents of the fifth year. Third-year residents perform low complexity surgery such as local anesthesia procedures and double J stent placement. Fourth-year residents perform mid complexity surgery such as simple prostatectomy and nephrostomies.

Medical training was analyzed by the difference in the volume of practical procedures, theoretical activities, and resident's workload.

## RESULTS

The outpatient clinic was reduced by 75.8% (1.466 vs. 6.049) of the average of previous years. The only data subgroup that remained similar to previous years was urology oncology new appointments, with a reduction of 28.1% (82 vs. 144) of the previous volume. The area affected the most was pediatric urology, with a reduction of 93.3% (4 vs. 60) of new appointments and 89.6% (26 vs. 251) of routine appointments. Table-2 shows full data of all types of outpatient appointments.

**Table 2:** Number of outpatient appointments and surgeries between April to July in 2020 compared to the average number of 2018 and 2019.

	NEW APPOINTMENTS		
	2018 and 2019*	**2020**	**%**
Urology Oncology	114	82	71.90
General Urology	166	11	6.60
Pediatric Urology	60	4	6.70
Kidney Transplant	103	14	13.60
	ROUTINE APPOINTMENTS		
	2018 and 2019*	**2020**	**%**
General Urology	5204	1294	24.90
Pediatric Urology	251	26	10.40
Kidney Transplant	151	35	23.20
	ALL APPOINTMENTS		
	2018 and 2019*	**2020**	**%**
Total	6049	1466	24.20
	SURGERIES		
	2018 and 2019*	**2020**	**%**
Cystoscopy	445.5	98	22.0
Double j placement	189	100	52.90
Prostate biopsy	114.5	46	40.20
Nephrostomy	89.5	55	61.50
Extracorporeal shockwave lithotripsy	68	4	5.90
Cystostomy	49	20	40.80
Postectomy	49	9	18.40
Transurethral resection of the prostate	48.5	9	18.6%
Percutaneous nephrolithotripsy	40	21	52.5%
Ureteroscopy	36	37	102.8%
Kidney transplant	35.5	6	16.9%
Radical prostatectomy	35	18	51.40
Simple prostatectomy	32	21	65.60
Urinary incontinence surgery	31	3	9.70
Laparoscopic radical nephrectomy	23	16	69.60
Laparoscopic partial nephrectomy	19.5	6	30.80
Graft urethroplasty	16.5	5	30.30
Vasectomy	15	4	26.70
Open radical nephrectomy	14.5	14	96.60
Orchiectomy	14	6	42.90
Urethroplasty	13.5	2	14.80
Cystolithotomy	11	3	27.30
Hidrocelectomy	10	1	10.0
Robotic radical prostatectomy	9.5	8	84.20
Vesico-ureteral reimplant	9.5	1	10.50
Bladder neck incision	8.5	3	35.30
Orchidopexy	8	1	12.50
Pyeloplasty	7.5	2	26.70
Urethrotomy	7.5	2	26.70
Radical cystectomy	7	5	71.40
Meatoplasty	7	1	14.30

* The average number of appointments and surgeries between April to July of 2018 and 2019.

Urodynamics exams were reduced to 88.0% of the average volume of previous years. In 2020, there were only 18 exams performed between April and July compared to 147 in 2018 and 152 in 2019.

**Figure 1:**
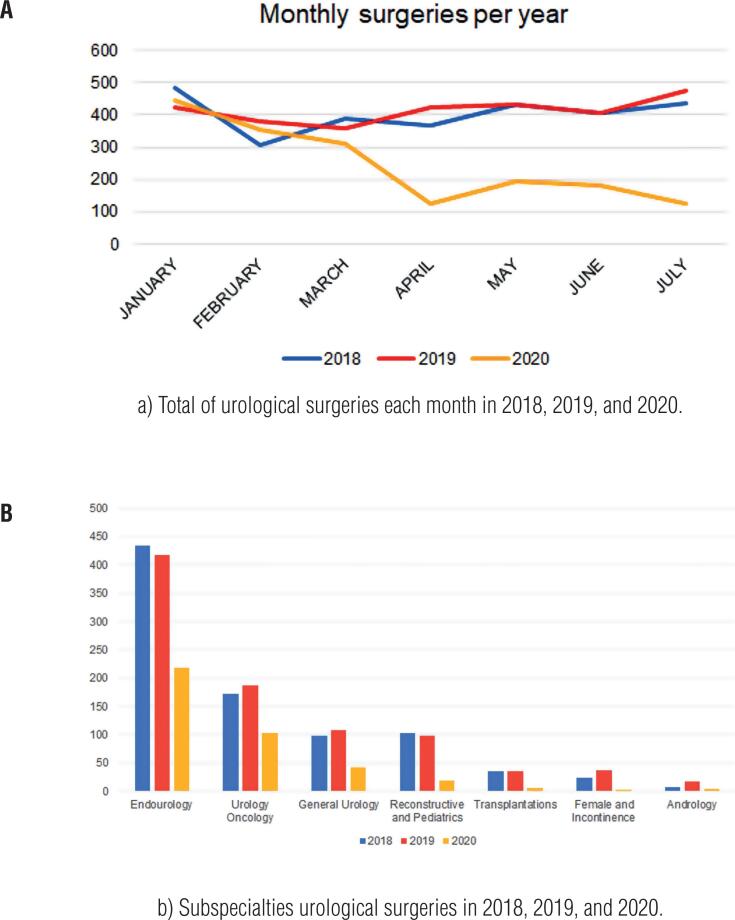
Urology surgeries in 2018, 2019, and 2020.

Elective surgeries were reduced by 63.4% of the average volume of previous years. Figure-1A shows the monthly reduction in this year compared to previous years. The most affected areas were Female/Incontinence with a reduction of 89.0%, Transplantations (83.1% reduction), and Reconstructive/Pediatrics (81.3% reduction). Oncology and Endourology were the two areas the least affected (42.4% and 48.7% respectively). Table-2 shows full data of all non-urgent surgeries.

The only majors surgeries that remained over 50% of the regular volume were open radical nephrectomy (100%, 14/14), robotic radical prostatectomy (84.2%, 8/9.5), open radical cystectomy (71.4%, 5/7), laparoscopic radical nephrectomy (69.6%, 16/23), simple prostatectomy (65.6%, 21/32), prostate biopsy (62.1%, 18/29) radical orchiectomy (57.1%, 4/7), open radical prostatectomy (51.4%, 18/35) and percutaneous nephrolithotripsy (50.0%, 17/34). Figure-1B shows complete data for all major surgeries.

Regarding medical training, outpatient appointments and surgeries were also reduced during all residency years. There was a reduction of 61.0% (146 vs. 477) procedures of fifth-year residents, 38.2% (140 vs. 227) of fourth-year residents and 61.0% (186 vs. 477) of third-year residents. Theoretical activities were doubled, from an average of 4 hours to 8 hours weekly. Dry lab activities (laparoscopic simulator) remained similar, with an average of 2 hours weekly per resident. The workload was reduced to 30 to 40% of previous years.

Hospital admissions were reduced by 54.6% (259/570), despite not having a great difference in hospital stay, with an average +0.81 days per patient (5.42 vs. 4.61), or in mortality rate (less than 1% in all years). Also, emergency consultations and admissions were not reduced, with 406 emergency urology patients in 2020, compared to an average of 370 in the same period in previous years.

## DISCUSSION

The COVID-19 has deeply changed urology practice, with a great reduction in outpatient appointments, exams, and surgeries. We developed a contingency plan that was similar to most other countries (2–5).

Our analysis includes a 4-month interval. We chose to start the analysis in April because the contingency protocol started on March 23th (and is still ongoing). We believe this period to be the most representative. Also, despite partial lockdown being stablished since early March, we are still having a high number of daily new cases. In May, Latin America was declared by the WHO the new epicenter of COVID-19, and, so far, the Brazilian peak of cases was in July and August.

It is important to note that there are subspecialties that were more affected during the urology pandemic. Incontinence, reconstructive, pediatrics urology and andrology were the most affected, with numbers near the 15% average volume and, in some cases, as low as zero.

The full impact this might have on public healthcare is still to be determined. Our hospital works with near 100% full capacity the whole year, but the patients that were not operated during this pandemic probably will have to be operated in the next months despite the overload capacity we normally work, postponing other surgeries in a cascade effect so we will have to think strategies to increase our regular surgical volume during the next few years to diminish the healthcare impact in our society. The use of definite pathways to access the hospital and telemedicine may be an effective strategy after the COVID-19 pandemic (6, 7).

We found a great reduction in outpatient clinics, urodynamics exams, elective surgeries, transplantation, and urgent surgeries. That is similar to other findings in the literature. Tan et al. reported that in a Residency Program in Singapore, elective surgeries were reduced by 70% within 2 months (8). A survey in Italy, the first Western country deeply affected by COVID-19, a survey showed that complete suppression of surgical training exposure might have been as high as 62.1% (9). In the US, another survey reported a significant reduction in surgical volume up to 83-100% varying by specialty (10). In Brazil, 83.2% and 89.6% of respondents of an online survey reported a reduction of over 50% of patients visits and elective surgeries (11).

As far as we know, this is the only paper that fully reports all outpatient clinics, exams, and surgical volume compared to previous years, as all other studies were based on online surveys and not hospital logs.

Regarding medical training, there is a great concern that residents may not fulfill the mandatory training requirements due to a reduction in the clinic and especially surgeries. A survey by Paesano et al. reported that 75% of respondents stated their surgical training has been completely affected and 65% stated the theoretical training was also affected (12).

Urology Residency in Brazil is organized as a 5-year residency, similar to most North American and European residencies. Also, it is important to note that most surgeries at our hospitals are performed by the fifth-year resident with a urologist staff as a supervisor. Therefore, the reduction in surgical volume is of bigger concern because last year residents did not perform the needed number of surgeries in the previous years and will be graduating in February 2021.

During the pandemic, we are performing an increased number of lectures and a weekly recorded surgery conference. Several institutions are adopting a similar approach to compensate for the reduction of residency activities. In a USA survey, 95% of respondents reported a transition to virtual education platforms (10). Porpiglia et al. state that the use of smart technologies should be maximized and implemented. This, indeed, may partially compensate for the reduction in surgical volume and clinical activity (13).

While we wait for a statement from the National Medical Residency Commission, we discuss possibilities to decrease the damage to the residency training, such as extending the end of residency (or even a whole year), extra fellowship positions to our residents at our department and/or increasing surgical volume after the pandemic.

## CONCLUSION

The reduction in urology volume - outpatient clinic, inpatient, and surgeries - during the COVID-19 was very high, especially in some areas such as reconstructive, pediatrics, and urogynecology. These areas, despite having a low number of high-priority procedures (no urgent or life-threatening procedures if postponed), have a great impact on patient wellbeing and quality of life, especially in an underdeveloped country with a public healthcare system that usually works in an overload capacity.

In the same way, the COVID-19 pandemic will severely affect urology residency training, especially the non-oncologic areas. The Brazilian situation is critical because of the high number of new cases still after four months of the pandemic, with no sign of resolution in the short-term. Therefore, it is mandatory to discuss strategies to train the residents during the pandemic.

## References

[1] 1. Heldwein FL, Loeb S, Wroclawski ML, Sridhar AN, Carneiro A, Lima FS, et al Teoh JY. A Systematic Review on Guidelines and Recommendations for Urology Standard of Care During the COVID-19 Pandemic. Eur Urol Focus. 2020; 6:1070-85. 10.1016/j.euf.2020.05.020 PMC7274599 32532703

[2] 2. Pang KH, Carrion DM, Rivas JG, Mantica G, Mattigk A, Pradere B, et al. The Impact of COVID-19 on European Health Care and Urology Trainees. Eur Urol. 2020; 78:6-8. 10.1016/j.eururo.2020.04.042 PMC7183959 32376133

[3] 3. de la Reza MT, Autrán-Gómez AM, Tardío GU, Bolaños JA, Rivero JCG. Emergency Surgery in Urology during the COVID-19 Pandemic. Int Braz J Urol. 2020; 46(suppl.1):201-206. 10.1590/S1677-5538.IBJU.2020.S125 PMC7719990 32618465

[4] 4. Rodríguez-Covarrubias F, Castillejos-Molina RA, Autrán-Gómez AM. Summary and considerations in genitourinary cancer patient care during the COVID-19 Pandemic. Int Braz J Urol. 2020; 46(suppl.1):98-103. 10.1590/S1677-5538.IBJU.2020.S115 PMC7719999 32549077

[5] 5. Carneiro A, Wroclawski ML, Nahar B, Soares A, Cardoso AP, Kim NJ, et al. Impact of the COVID-19 Pandemic on the Urologist's clinical practice in Brazil: a management guideline proposal for low- and middle-income countries during the crisis period. Int Braz J Urol. 2020; 46:501-10. 10.1590/S1677-5538.IBJU.2020.04.03 PMC7239291 32271512

[6] 6. Esperto F, Prata F, Civitella A, Pang KH, Marchioni M, Tuzzolo P, et al. Implementation and strategies to ensure adequate coordination within a Urology Department during the COVID-19 pandemic. Int Braz J Urol. 2020; 46(suppl.1):170-80. 10.1590/S1677-5538.IBJU.2020.S122 PMC7719985 32649082

[7] 7. Cacciamani GE, Shah M, Yip W, Abreu A, Park D, Fuchs G. Impact of Covid-19 on the urology service in United States: perspectives and strategies to face a Pandemic. Int Braz J Urol. 2020; 46(suppl.1):207-14. 10.1590/S1677-5538.IBJU.2020.S126 PMC7720000 32618466

[8] 8. Tan YQ, Wang Z, Tiong HY, Chiong E. The Good, the Bad, and the Ugly of the COVID-19 Pandemic in a Urology Residency Program in Singapore. Urology. 2020; 142:244-5. 10.1016/j.urology.2020.05.027 PMC7256528 32473937

[9] 9. Amparore D, Claps F, Cacciamani GE, Esperto F, Fiori C, Liguori G, et al. Impact of the COVID-19 pandemic on urology residency training in Italy. Minerva Urol Nefrol. 2020; 72:505-9. 10.23736/S0393-2249.20.03868-0 32253371

[10] 10. Fero KE, Weinberger JM, Lerman S, Bergman J. Perceived Impact of Urologic Surgery Training Program Modifications due to COVID-19 in the United States. Urology. 2020; 143:62-7. 10.1016/j.urology.2020.05.051 PMC7274971 32512110

[11] 11. Gomes CM, Favorito LA, Henriques JVT, Canalini AF, Anzolch KMJ, de Carvalho Fernandes R, et al. Impact of COVID-19 on clinical practice, income, health and lifestyle behavior of Brazilian urologists. Int Braz J Urol. 2020; 46:1042-71. 10.1590/S1677-5538.IBJU.2020.99.15 PMC7527096 32539253

[12] 12. Paesano N, Santomil F, Tobia I. Impact of COVID-19 Pandemic on Ibero-American Urology Residents: Perspective of American Confederation of Urology (CAU). Int Braz J Urol. 2020; 46(suppl.1):165-9. 10.1590/S1677-5538.IBJU.2020.S120 PMC7719988 32550707

[13] 13. Porpiglia F, Checcucci E, Amparore D, Verri P, Campi R, Claps F, et. al. Slowdown of urology residents’ learning curve during the COVID-19 emergency. BJU Int. 2020; 125:E15-E17. 10.1111/bju.15076 PMC7262049 32274879

